# Limitations of Blood Pressure Measurements in Pediatric Trauma Patients During Field Triage

**DOI:** 10.7759/cureus.70084

**Published:** 2024-09-24

**Authors:** Amy Frederick, Jason Winslow, Vinci Jones, Lauren Rothburd, Briana Florez, Ellen Van Auken, Heather Reens, Theresa Drucker, Idamis Melendez Vassall, Anupreet Kaur, Amirun Mahia, Sarah Eckardt, Catherine Caronia, Patricia A Eckardt

**Affiliations:** 1 Trauma, Good Samaritan University Hospital, West Islip, USA; 2 Emergency Medicine, Suffolk County Department of Health Services, Yaphank, USA; 3 Pediatric Surgery, Good Samaritan University Hospital, West Islip, USA; 4 Nursing, Good Samaritan University Hospital, West Islip, USA; 5 Nursing, Molloy University, Rockville, USA; 6 S Jay Levy Fellowship Program, City University of New York, New York City, USA; 7 Data Scientist, Eckardt & Eckardt Consulting, St. James, USA; 8 Pediatrics, Good Samaritan University Hospital, West Islip, USA

**Keywords:** blood pressure, vital signs, emergency medical services (ems), field triage, trauma pediatric

## Abstract

Introduction: Recent revisions of national field triage guidelines recommend the addition of age-specific systolic blood pressure (SBP) measurement for identifying the most severely injured children requiring transport to a trauma center. The purpose of this study was to determine the frequency in which blood pressures are documented by Emergency Medical Service (EMS) providers and the role this measurement has had, among other factors, in triage decisions.

Methods: This is an exploratory descriptive study with a retrospective review from the trauma registry database of all pediatric trauma admissions that arrived by EMS at a level II pediatric trauma center from January 1, 2019 to December 31, 2022.

Results: Two hundred ninety-eight patient records of patients aged 0 to 14 were included. EMS providers documented blood pressure in 70.1% of the total sample. A significant difference in the frequency of this documentation was seen between ages zero to nine and = > 10 years (χ^2^(1,298) = 28.98 p <0.001). No children ages zero to nine years had SBP of < 70 mmHg + (2x age in years) documented by EMS. There were two children aged = > 10 who had a documented SBP < 90 and 12 children with documented EMS heart rate > SBP.

Conclusion: Many children transported by EMS in this hospital’s catchment area did have a field blood pressure measurement documented, but the frequency was significantly less in younger-aged children. The blood pressure measurements of children determined to have severe injuries in the sample did not meet the inclusion criteria for high risk of serious injury by the newly established national guidelines. This suggests other prehospital criteria, such as mechanism of injury or visual cues, prompted EMS to transport these pediatric trauma patients to a regional trauma center for specialized care.

## Introduction

In 2021, the “National Guidelines for the Field Triage of Injured Patients” were revised by a multidisciplinary expert panel led by the American College of Surgeons Committee on Trauma (ACS-COT) after a systematic review of the most recent evidence-based literature [1]. These pre-hospital triage guidelines are used for accurate and timely triage by Emergency Medical Services (EMS) to identify the most seriously injured patients in need of a specialized trauma center to provide optimal care and reduce patient morbidity and mortality. Field triage guidelines include physiologic and mechanical criteria demonstrating high predictive value for severe injury that serves as the basis for EMS protocols [2].

Although physiologic criteria are known predictors of injury severity, previous national triage guidelines did not include age-specific criteria for systolic blood pressure (SBP) and heart rate (HR) for ages 0-14 years. Reasons identified in prior studies include the acceptance of overtriage in children, barriers to obtaining accurate readings, and difficulty improving rates of documented blood pressure by pre-hospital providers [3]. Additionally, lack of practice in general pediatric skills and anxiety working with children have been reasons noted among EMS providers as barriers to obtaining pediatric BPs [4]. These barriers and factors may result in the inability of some field triage protocols to identify children in need of specialized trauma care when using blood pressure measurement as the foremost triage criteria.

However, current evidence supports the addition of age-specific physiologic criteria including SBP and HR measurements to field triage criteria, and accordingly, this was added to the 2021 revisions. The recommendations were grouped by ages (in years) of zero to nine, 10 to 64, and 65 and older with cited evidence-based studies listing physiologic criteria assessment first as age-adjusted hypotension is an independent predictor of mortality [5]. This was further supported in a 2023 study looking at the association of omitted vital sign measurements and patient outcomes which concluded that patients without any blood pressure measurements in the field had a higher mortality rate of 4.3% vs. 1.1 (P < 0.001%) [6]. Additional evidence for the value of blood pressure (BP) and HR assessment is also noted in the Pediatric Advanced Life Support (PALS) curriculum which reflects the latest science and education [7]. 

Missing vital sign data have been found in multiple studies. Additionally, the challenge in obtaining pre-hospital vital signs and determining accuracy has been recognized for many years. A retrospective study examining 2,081 patient visits to the Emergency Department (ED) found that documentation of temperature was missing from over 300 charts of the sample (approximately 16%) [8]. Missing data were higher for HR, respiratory rate (RR), BP, and pulse oximetry (approximately 42%, 60%, 69%, and 74%, respectively). Moreover, the study identified rates of missing vital signs and their predictive factors. Variables such as acuity of the patient’s condition and shift of presentation (day vs. night shift) were found to be predictive factors of incomplete vital sign obtainment. Further found in the literature, a 2023 study was published examining the records of 1,929 pediatric trauma patients treated at a single-site level I pediatric trauma center [9]. Results indicated 37.6% of charts to be missing at least one value and 7.8% of charts to be missing any value. SBP, HR, RR, oxygen saturation, and GCS were found to be missing in 21.7%, 11.1%, 22.2%, 19.4%, and 9% of charts, respectively. Recently, a 2024 retrospective observational study evaluated challenges in prehospital assessment for pediatric trauma patients [10]. This study found that 81.8% of children in the total sample (440 children) were assessed, and a complete set of vital signs was obtained in only 29.3% of the sample. Additionally, in 2017, a multi-center study of 5,594 children ages 0-15 found that 29% of pre-hospital records were missing vital signs [11]. Glasgow Coma Score (GCS) was not obtained by EMS in 4% of cases. Abnormal RR was not obtained by EMS in 5% of cases. SBP was not obtained by EMS in 20% of cases. When the frequency of pre-hospital vital sign documentation was compared by age group, the data showed the lowest frequency in the youngest children, with 79% of children < 1-year missing vital signs in contrast to 2% of children 10-15 years old. Lastly, in those children with a documented BP, there was only a 19% agreement between ED vital signs and first documented prehospital vital signs, but the significance of this is unclear [11]. Additional evidence from a National EMS Information System (NEMSIS) study found that 61.5% of pediatric EMS transports had at least one abnormal vital sign and highly variable records with incomplete documentation [12]. Nevertheless, by analyzing the frequency and accuracy of SBP obtainment in the pediatric trauma population by EMS, we can assess where gaps remain and determine additional field triage criteria to use in order to identify a child requiring the resources of a trauma center.

Therefore, the purpose of this study was to determine the frequency that blood pressure documented by EMS providers and the role this measurement has had, among other factors, in triage decisions. Additionally, other prehospital criteria were explored which may have prompted EMS to transport these pediatric trauma patients to a regional trauma center for specialized care. 

## Materials and methods

Design

This was an exploratory, non-interventional, descriptive, single-site retrospective review from the trauma registry database of all pediatric trauma admissions, ages 0-14, that arrived by EMS to a level II pediatric trauma center. The data collection period was from January 1, 2019 to December 31, 2022. The study design was determined to be exempt by the Good Samaritan University Hospital Office of the Institutional Review Board on June 20, 2023. STROBE guidelines from the EQUATOR network for reporting observational studies were used to ensure proper reporting [13].

Setting

The site of this study took place at a suburban academic medical center that served, at the time, as an ACS-verified level II adult and pediatric trauma center, located in Suffolk County, New York. The hospital observes over 80,000 ED patient visits per year, of which 18,000 are pediatric. The hospital is served by a hybrid EMS system that is comprised of paid and volunteer providers from ambulance companies and fire departments. More than 22,000 EMS-transported patients, of which over 1,700 are pediatric, are brought to this facility per year.

Population

The sample was comprised of all pediatric trauma admissions, ages 0 to 14, who arrived at the facility via EMS from January 2019 to December 2022. This population consists of injured children who required admission, treatment, and/or surgery at the pediatric trauma center and met inclusion criteria for submission to the National Trauma Data Bank.

Data collection and handling

The following variables were extracted from the trauma registry: trauma registry number, medical record number, arrival date, arrival time, age, trauma type (blunt, penetrating, thermal; drowning not considered trauma in registry), ICD-10 number, transport mode, EMS BP documented, EMS SBP, EMS pulse rate documented, EMS pulse rate, preactivation, activation level, ED arrival injury severity score (ISS), ED arrival SBP documented, ED arrival pulse rate documented, and ED arrival pulse rate.

The patients were grouped into two age groups: 0-9 years and 10-14 years. These are the age groups for which the National Guidelines for the Field Triage of Injured Patients sort pediatric trauma patients [1]. As part of the Red Criteria, children nine years old and younger are considered to have a serious injury if their SBP is less than 70mm Hg + (2x age in years). The criteria also states that children 10-14 years of age are considered to have serious injury if their SBP is less than 90 mmHg. Additionally, for children ages 10-14 years, HR > SBP is also an indicator of serious injury [1]. Therefore, the data were then coded to examine if the SBP was documented to be less than 70 mmHg + (2x age in years) for children = < 10 years of age. For children => 10 years of age, documented SBP was also coded to examine if SBP < 90 mmHg or SBP < HR. These two groups were assessed to see if there was a significant difference between the documentation of SBP at the pediatric trauma center and the EMS SBP (if present). Additionally, recorded BP was examined as a useful predictor in identifying seriously injured children. Lastly, other factors were explored that may have prompted EMS to transport these pediatric trauma patients to a regional center.

Statistical analysis

Data were extracted from the trauma registry database and exported for analysis into IBM Statistical Package for the Social Sciences (SPSS) Statistics for Windows, version 28 (IBM Corp., Armonk, NY, USA) and Stata Statistical Software: Release 17 (StataCorp LLC, College Station, TX). Though the objectives of the study are descriptive, overall variation in SBP and HR measurements included inferential analyses. For inferential analyses, a two-tailed testing approach with a chosen significance level of p < 0.05 was used. Independent sample t-tests and one-way ANOVAs were used to compare groups with continuous outcomes. The chi-square test for Independence and Fischer’s exact test (FET) was conducted to analyze categorical data if more than 20% of cells had expected counts less than 5. Power was sufficient with a minimum sample size of n=102. In addition to inferential estimates, inter-rater reliability, and Cohen Kappa statistics were estimated on documented SBP for EMS and ED nursing staff.

## Results

The final sample abstracted from the trauma registry database for pediatric patients who met the inclusion criteria was n = 298. The majority of patients were less than 10 years of age (n=160, 53.69%), though not largely different than patients greater than or equal to age 10 (n=138, 46.31%). Results indicated that patients in this sample arrived mostly during the day shift between the hours of 0700 and 1500 (n=192, 64.43%). Additionally, the majority of the types of traumas were blunt (n=287, 96.31%). Patients also arrived predominantly by ambulance (n=296, 99.33%). The sample of patients did not have preactivation (n=247, 83.45%), and no activation once admitted to the ED (n=105, 35.23%), along with an ED arrival ISS considered “mild” (n=263, 91.96%).

EMS documented SBP on the total sample 70.1% of the time (Table 1). The frequency of EMS SBP documentation was significantly different (χ2(1,298) = 28.98 p < 0.001) by age group, 0-9 years vs. 10-14 years (57% and 85.5%, respectively) (Tables 2, 3). ED nurses documented a BP on children in the sample 92.7% of the time with a significant difference (χ2(1,298) = 13.23 p < 0.001) by age group, 0-9 years vs. 10-14 years (87.5% and 98.6%, respectively). The frequency of BPs documented by ED nurses was significantly higher compared to EMS (χ2(1,298) = 50.72 p < 0.001) (70.1% and 92.7%, respectively).

**Table 1:** Descriptive characteristics of total sample (n = 298) ED = emergency department. EMS = emergency medical services. BP = blood pressure. SBP = systolic blood pressure. ^a^Mean, standard deviation (SD). ^b^Frequency, percentage (%).

Characteristic	Univariate statistic
Age^a^	8.22 (4.53)
Year of trauma^b^	
2019	65 (21.6%)
2020	70 (23.3%)
2021	77 (25.6%)
2022	89 (29.6%)
Shift arrived in ED^b^	
0700-1500 hours	22 (7.2%)
1501-2300 hours	84 (28.2%)
2301-0659 hours	192 (64.4%)
Trauma type^b^	
Blunt	287 (96.4%)
Burns	5 (1.6%)
Penetrating	6 (2.0%)
Transport mode^b^	
Ambulance	296 (97.0%)
Police	2 (3.0%)
EMS BP documented^b^	
Yes	211 (70.1%)
No	90 (29.9%)
EMS SBP^a^	123.5 (19.9)
EMS pulse rate documented^b^	
Yes	237 (77.7%)
No	68 (22.3%)
EMS pulse rate^a^	105.1 (21.06)
Preactivation^b^	
Yes	49 (46.4%)
No	247 (53.6%)
Activation level^b^	
Code T	28 (9.4%)
Trauma alert	86 (28.9%)
Consult	79 (26.5%)
None	105 (35.2%)
ED arrival injury severity score (ISS)^a^	5.0 (0.34)
ED arrival ISS category^b^	
Mild (0-9)	263 (92.0%)
Moderate (10-15)	13 (4.5%)
Severe (16-24)	7 (2.4%)
Profound (=>25)	3 (1.0%)
ED arrival SBP documented^b^	
Yes	279 (92.7%)
No	22 (7.3%)
ED arrival SBP^a^	119.13 (17.12)
ED arrival pulse rate documented^b^	
Yes	294 (96.4%)
No	11 (3.6%)
ED arrival pulse rate^a^	108.47 (26.71)

**Table 2:** Comparison of variables between children aged < 10 years and children aged => 10 years (n= 298) ED = emergency department. EMS = emergency medical services. BP = blood pressure. SBP = systolic blood pressure. ^a^Mean, standard deviation (SD). ^b^Frequency, percentage (%). ^c^Independent sample t-tests were conducted for continuous variable estimates, crosstabulation chi-square estimates were conducted for categorical data (except when expected count < 5 in a cell Fisher’s exact statistic was estimated).^d^Inferential statistic not calculated. *Significant p-value <0.05. **Significant p-value <0.01. ***Significant p-value <0.001.

Characteristic	Age < 10 years (n=160)	Age =>10 years (n=138)	P-value^c,d^
Age^a^	5.49 (2.51)	12.54 (1.29)	--^d^
Year of trauma^b^			0.531
2019	38 (23.8%)	25 (18.1%)	
2020	38 (23.8%)	31 (22.5%)	
2021	41 (25.6%)	36 (26.1%)	
2022	43 (29.6%)	46 (33.3%)	
Shift arrived in ED^b^			0.015*
0700-1500 hours	115 (71.9%)	77 (55.8%)	
1501-2300 hours	35 (21.9%)	49 (35.5%)	
2301-0659 hours	10 (6.3%)	12 (8.7%)	
Trauma type^b^			0.579
Blunt	155 (96.9%)	132 (95.7%)	
Burns	3 (1.9%)	2 (1.4%)	
Penetrating	2 (1.3%)	4 (2.9%)	
Transport mode^b^			0.501
Ambulance	158 (98.8%)	138 (100.0%)	
Police	2 (1.3%)	0 (0.0%)	
EMS BP documented^b^			< 0.001***
Yes	91 (56.9%)	118 (85.5%)	
No	69 (43.1%)	20 (14.5%)	
EMS SBP^a^	120.7 (20.44)	125.6 (19.5)	0.042*
EMS pulse rate documented^b^			0.008**
Yes	118 (73.8%)	119 (86.2%)	
No	42 (26.3%)	19 (13.8%)	
EMS pulse rate^a^	110.21(18.72)	96.63 (19.38)	< 0.001***
Preactivation^b^			0.159
Yes	22 (13.8%)	27 (19.9%)	
No	138 (86.3%)	109 (80.1%)	
Activation level^b^			0.792
Code T	13 (8.1%)	15 (10.9%)	
Trauma alert	47 (29.4%)	39 (28.3%)	
Consult	45 (28.1%)	34 (24.6%)	
None	55 (34.4%)	50 (36.2%)	
ED arrival injury severity score (ISS)^a^	5.2 (4.74)	5.4 (4.9)	0.479
ED arrival ISS category^b^			0.910
Mild (0-9)	142 (92.2%)	121 (91.7%)	
Moderate (10-15)	6 (3.9%)	7 (5.3%)	
Severe (16-24)	4 (2.6%)	3 (2.3%)	
Profound (=>25)	2 (1.3%)	1 (0.8%)	
ED arrival SBP documented^b^			< 0.001***
Yes	140 (87.5%)	136 (98.6%)	
No	20 (12.5%)	2 (1.4%)	
ED arrival SBP^a^	116.29 (15.27)	123.65 (16.71)	< 0.001***
ED arrival pulse rate documented^b^			0.775
Yes	157 (98.1%)	136 (98.6%)	
No	3 (1.9%)	2 (1.4%)	
ED arrival pulse rate^a^	111.91 (22.87)	94.96 (19.45)	< 0.001***

**Table 3:** Comparisons between provider type and age groups EMS = emergency medical services. ED = emergency department. SBP = systolic blood pressure. ^a^Significance tests for continuous and categorical variables were two-tailed independent samples t-test, and a cross-tabulation two-tailed chi square estimate with 1 degree of freedom (Fisher’s exact estimate for cells with expected value 5 or less), respectively. ^b^Percentage within group. ^c^SD = Standard Deviation. *Significant p-value <0.05. ***Significant p-value <0.001.

Outcome	Group		P-value^a^
	EMS provider	ED nursing provider	
Children < 10 years of age			
SBP documented (%)^b^	91 (56.9%)	140 (87.5%)	< 0.001***
Mean SBP (SD)^c^	120.7 (20.44)	116.29 (15.27)	0.029*
Pulse rate documented (%)^b^	118 (73.8%)	157 (98.1%)	< 0.001***
Mean pulse rate (SD)^c ^	110.21 (18.72)	111.91 (22.87)	0.67
Children => 10 years of age			
SBP documented (%)^b^	118 (85.5%)	136 (98.6%)	< 0.001***
Mean SBP (SD)^c^	125.6 (19.5)	123.65 (16.71)	0.337
Pulse rate documented (%)^b^	119 (86.2%)	136 (98.6%)	< 0.001***
Mean pulse rate (SD)^c ^	96.63 (19.38)	94.96 (19.45)	0.442

**Figure 1:**
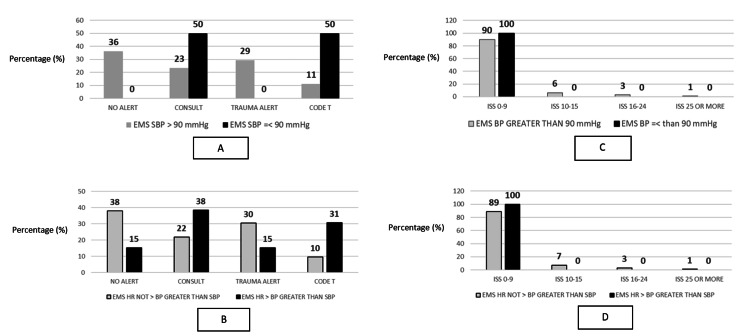
Systolic blood pressure red criteria 10 and older across ISS category and trauma activation level (A) Percentage of patients (=> 10 years of age) with EMS SBP stratified by BP>90 (n=116) or =<90 (n=2) and trauma activation level. (B) Percentage of patients (=> 10 years of age) stratified by EMS SBP (n=105) and EMS HR > EMS SBP (n=13) and trauma activation level. (C) Percentage of patients (=>10 years of age) with EMS SBP stratified by BP>90 (n=110) or <90 (n=2) and ISS score by category. (D) Percentage of patients (=> 10 years of age) stratified by EMS HR not > EMS SBP (n=100) and EMS HR > EMS SBP (n=12) and ISS. EMS = emergency medical services. SBP = systolic blood pressure. BP = blood pressure. HR = heart rate. ISS = injury severity score.

There was low inter-rater agreement (0.37, 95% CI 0.24;.48) and Kappa estimate (0.28, 95% CI 0.20;0.36) between the EMS and the ED-documented measurement of BP in the sample. For children => 10 years of age, the documented BP by EMS was not within the parameters of the 2021 “National Guideline for the Field Triage of Injured Patients” criteria for identifying seriously injured children for any patient (0.0%). As there were no children in the < 10 years of age group that had SBP of < 70 mmHg + (2x age in years), the Red Criteria of SBP < 70 mmHg + (2x age in years) was not calculated. Using an SBP of less than 90 mmHg or an HR>SBP as field triage criteria alone in patients older than 10 years was not associated with EMS trauma activation level or ISS of patients (Figures 1A-1D).

Of the two patients 10 years of age and older with an EMS SBP < 90 mmHg, one (50%) had the highest-level activation of “Code T,” while the other patient (50%) was a trauma consult (lowest level of trauma notification) in the ED. In contrast, 13 patients (11%) with an EMS SBP > 90 mmHg had “Code T” activation, and 27 (23%) had a trauma consult. The percentage of patients with an ISS score of 0-9 for patients with an EMS SBP < 90 mmHg and EMS SBP => 90 mmHg was 100% and 90%, respectively. The only patient in the sample with an ISS of 25 or greater did not have an EMS SBP < 90 mmHg. Four patients (31%) with an EMS HR > EMS SBP had a “Code T” notification, while the 10 patients (10%) without an EMS HR > EMS SBP had a “Code T” notification. “Trauma Alert” (second-level activation) notifications did not occur for two (15%) and 40 (38%) patients with an EMS HR > EMS SBP and those without an EMS HR > EMS SBP, respectively. Twelve patients (100%) with an EMS HR > EMS SBP had an ISS score of 0-9 compared to 100 patients (89%) without an EMS HR > EMS SBP with an ISS score of 0-9.

## Discussion

This study found that EMS documented field BP and HR for pediatric trauma patients aged <15 years, but the frequency was different when divided into two age groups. Compared to the frequency of all the children in the study, the number of recorded BP measurements increased in children aged = >10 years and decreased in patients aged < 10 years. Although the population was small in our review, the results were consistent with larger-scale studies confirming that the younger the patient, the lower the probability of documented BP. Studies in diverse populations, nationally and internationally, also noted similar results and rationale for the lack of BP measurement [11]. The obstacles mentioned include inexperience with children, unavailability of age-appropriate equipment, environment, and difficulty assessing crying and injured children. Additional findings in the study also correlated with larger studies including the variability in EMS and ED nurse BP recordings showing that the increase in frequency of BP measurement documented by nurses increases as the child’s age increases [14,15].

The literature supports EMS assessment of BP in children for determining injury severity and triage to the appropriate trauma center. The mentioned barriers to obtaining accurate readings in young children and variable results noted by providers support the value of additional criteria for pre-hospital triage. Pre-hospital providers may also look at alternate assessment tools to ensure they account for missing vital signs and/or utilize other indicators that can identify children who are severely injured when the BP measurement is not available or may be unreliable [11]. Indicators, such as age and location of injury, may more so automatically prompt EMS to transfer to a particular level of care destination. A study looking at triage decisions between pediatric age groups found that younger patients were more likely to be brought to a trauma center; especially those with thorax or abdominal injuries [16]. Moreover, pediatric patients with spinal, head, and thorax injuries were found to have the highest likelihood of transfer to a trauma center. As we discovered in our study BP was less frequently documented in younger children, injury site, type, or age may pre-emptively factor into the transport decision when assessing a younger pediatric patient, and BP measurement is not available.

Interestingly, another study looked at overall EMS judgment in trauma center transportation decisions [17]. Three standardized and widely accepted scoring systems were compared to EMS judgment of mortality (on a four-point scale), and it was found that EMS’ prediction scored as accurately as the scoring systems. The same was revealed for EMS’ prediction of patients who died or required emergent surgical intervention. On the other hand, a later study critically reviewed the literature on the topic of paramedic judgment in trauma transport decisions and found unclear support for its accuracy in triage as an independent method [18]. While EMS judgment as a standalone method may not be empirically supported, it is remiss to not mention the more likely possibility of its incorporation alongside defined and physiologic methods of triage. The conjunction of both physiologic and additional clinical signs observed by EMS during triage could very well contribute to the decision for the appropriate transport destination. While a meaningful and insightful piece of literature, it should also be mentioned that this study had major limitations; of note, comparing a multitude of institutions with varying clinical definitions, data collection methods, levels of completed data, paramedic experience, etc.

Additional triage tools for consideration include the Pediatric Assessment Triangle (PAT), a widely taught tool used by EMS providers, as well as ED clinician assessment for rapid evaluation of a child’s clinical status [15,19]. As evidence supports field BP, experts also looked at what would increase the frequency and accuracy of a BP measurement by EMS in children. One study looked at the impact of mandatory education for pre-hospital providers specific to pediatric assessment in children < 8 years and found that it led to a more thorough examination of the pediatric patient and contributed to an increase in documented vital signs [14]. We recognized the number of severely injured patients (ISS >15) in our study was small and limited, leading us to conclude that a larger sample must be analyzed for any inference on SBP as a reliable indicator to predict injury severity. Despite this, the data were useful in evaluating this trauma center’s over and under triage rates, current trauma activation guidelines, and opportunities to improve EMS and ED triage in the catchment area. The results of our limited patient number in this study showed SBP and HR in the severely injured (ISS>15) children did not correspond with the Red Criteria SBP or HR measurements of the 2021 “National Guidelines for the Field Triage of Injured Patients” for identifying severely injured children. None of the children => 10 years of age had a documented BP that met the criteria, and there were no children in the < 10 years of age group that had SBP of < 70 mmHg + (2x age in years). One patient with an ISS > 15 had a normal SBP in the field and a SBP documented by the ED nurse, which met the Red Criteria. We also looked at the patients in the study where a “Code T” was activated to see what factors besides BP contributed to the decision by EMS or ED staff that this patient was at high risk for serious injury. Of the 28 “Code T” activations, 21 were activated prior to the patient’s arrival based on the EMS report to the ED team en route to the hospital. It appears that visual cues, which have been cited in existing literature to play a potentially important role during field triage, or the mechanism of injury, including “pedestrian struck” or “MVC with significant damage,” contributed to the decision when physiologic assessment was normal [20]. All of the cases were reviewed by the trauma center’s pediatric trauma coordinator to determine if the highest-level activation was appropriate and identify opportunities for improvement.

This study intended to look at historical and present data of documented prehospital BP, variation between field and ED BP, and to determine if SBP is a reliable indicator for seriously injured patients and their transport destination. Results demonstrated that the EMS documentation of field BP is significantly less frequent in younger children <10 years of age. It also demonstrated that in all children aged < 15 years who were seriously injured, so much as to require admission to a pediatric trauma center, SBP was not a primary factor that EMS used to determine the need for transport to a trauma center.

Limitations

This was a single-site observational study that may not have results that are generalizable across other EMS settings as this retrospective review was limited to this hospital’s catchment area, and data were not population-based. In our sample, the ISS average was low, indicating the majority of this sample was not severely injured. Additionally, our sample size was small for children who met the Red Criteria: none for children 0-9 years of age, for children 10 years of age and older only two had an EMS SBP < 90 mmHg and 12 had an EMS HR > EMS SBP, limiting inference regarding field triage and the new guidelines. Additionally, as there was a time lag between EMS SBP and ED SBP recordings, inter-rater agreement estimates were limited by temporal discordance.

## Conclusions

In this study of 298 injured children, none of the seriously injured children in the population met the new SBP and/or HR Red Criteria in the National Guidelines for the Field Triage of Injured Patients. Other factors, such as the mechanism of injury or visual cues, in addition to SBP and HR, likely contributed to the determination made by EMS regarding the need to transport patients to a pediatric trauma center. These other factors should be a focus of future EMS education projects and research studies.
